# The Challenges and Opportunities of Pharmacoepidemiology in Bone Diseases

**DOI:** 10.1002/jbm4.10051

**Published:** 2018-04-30

**Authors:** Dunia Alarkawi, M Sanni Ali, Dana Bliuc, Jacqueline R Center, Daniel Prieto‐Alhambra

**Affiliations:** ^1^ Bone Biology Division Garvan Institute of Medical Research School of Medicine University of New South Wales Sydney Australia; ^2^ Centre for Statistics in Medicine and Nuffield Department of Orthopaedics Rheumatology and Musculoskeletal Sciences (NDORMS) University of Oxford Oxford UK; ^3^ Clinical School St Vincent's Hospital Sydney Australia; ^4^ MRC Lifecourse Epidemiology Unit Southampton UK; ^5^ GREMPAL Research Group (Idiap Jordi Gol Primary Care Research Institute) and CIBERFes Universitat Autonoma de Barcelona Barcelona Spain

**Keywords:** EPIDEMIOLOGY, GENERAL POPULATION STUDIES, STATISTICAL METHODS, OSTEOPOROSIS

## Abstract

Pharmacoepidemiology is used extensively in osteoporosis research and involves the study of the use and effects of drugs in large numbers of people. Randomized controlled trials are considered the gold standard in assessing treatment efficacy and safety. However, their results can have limited external validity when applied to day‐to‐day patients. Pharmacoepidemiological studies aim to assess the effect/s of treatments in actual practice conditions, but they are limited by the quality, completeness, and inherent bias due to confounding. Sources of information include prospectively collected (primary) as well as readily available routinely collected (secondary) (eg, electronic medical records, administrative/claims databases) data. Although the former enable the collection of ad hoc measurements, the latter provide a unique opportunity for the study of large representative populations and for the assessment of rare events at relatively low cost. Observational cohort and case‐control studies, the most commonly implemented study designs in pharmacoepidemiology, each have their strengths and limitations. However, the choice of the study design depends on the research question that needs to be answered. Despite the many advantages of observational studies, they also have limitations. First, missing data is a common issue in routine data, frequently dealt with using multiple imputation. Second, confounding by indication arises because of the lack of randomization; multivariable regression and more specific techniques such as propensity scores (adjustment, matching, stratification, trimming, or weighting) are used to minimize such biases. In addition, immortal time bias (time period during which a subject is artefactually event‐free by study design) and time‐varying confounding (patient characteristics changing over time) are other types of biases usually accounted for using time‐dependent modeling. Finally, residual “uncontrolled” confounding is difficult to assess, and hence to account for it, sensitivity analyses and specific methods (eg, instrumental variables) should be considered. © 2018 The Authors *JBMR Plus* published by Wiley Periodicals, Inc. on behalf of American Society for Bone and Mineral Research.

## Introduction

The field of pharmacoepidemiology (PE) was developed to enable the study of drug adverse events in the wider populations and to emphasize the importance of well‐designed research to characterize the utilization and effects of drugs when used in actual practice and in the community.^1^ PE applies epidemiologic methods to clinical pharmacology to provide an estimate of the probability of beneficial or adverse effects of a treatment in populations.^2^ Health care professionals, policy makers, and patients usually seek the highest level of information about the effects of treatments. Nevertheless, it is estimated that more than half of medical treatments lack valid evidence of effectiveness, particularly for long‐term and patient‐centered outcomes.^3^, ^4^

Similar to other clinical research, the selection of the study design for PE studies depends on the research question. Randomized controlled trials (RCTs) are considered the gold standard for providing the highest level of evidence about the efficacy and safety of treatments. In osteoporosis research, numerous high‐quality RCTs have been conducted to assess the efficacy (under ideal and controlled circumstances) and safety (in restricted populations) of anti‐osteoporosis medications.^5^, ^6^, ^7^, ^8^, ^9^ Despite the strengths of those studies, they have their limitations and their results do not reflect the true effects of anti‐osteoporosis treatments in real‐world patients and actual practice settings. On the other hand, observational studies, using large data sets to study the effectiveness (under real‐world conditions) of these same medications (once marketed), have been conducted extensively in osteoporosis research, and their findings complement those from RCTs.^10^, ^11^, ^12^, ^13^ With longer follow‐up, the inclusion of more complex and older patients, and larger patient numbers, observational studies can, under certain assumptions and when properly conducted and analyzed, identify clinically important effects and study rare outcomes better than RCTs.^(14)^ Hence, the use of such studies for post‐marketing surveillance as recommended by drug regulatory agencies.^15^ Nevertheless, observational studies have a lot of challenges, including bias and issues with completeness and validity resulting from the nature of these data, and the conditions under which they are collected. Careful framing of the research question with appropriate study design and application of statistical analysis techniques can yield findings with validity and improve our understanding of treatment effects.^16^

This review discusses (with a focus on anti‐osteoporosis treatments) the differences between RCTs and observational studies in the study of drug effects. It summarizes the types of data sources used and the common study designs implemented in pharmacoepidemiology and explores the opportunities of such data, including the breadth of information sources and their inherent challenges and how to deal with them.

## Randomized Controlled Trials and Observational Studies

RCTs are the main method for evaluating the efficacy and safety of treatments. They are conducted under highly controlled conditions to ensure high internal validity and compliance, thus ensuring that differences in outcomes can solely be attributed to differences between the drug and placebo.^17^ Although RCTs have a lot of advantages, their design limits their ability to provide answers about issues commonly encountered by clinicians in real‐world patient settings (Table 1). It has been reported that 50%^18^ to 80% of patients receiving treatment for osteoporosis would not be eligible for a randomized controlled trial because of comorbidities, previous treatment with bone‐active agents, or the use of other medications.^19^ Therefore, results from RCTs may have limited generalizability to the general patient population. Furthermore, because RCTs have a limited duration of follow‐up time (commonly 3 years) with a restricted sample size, their ability to identify rare or long‐term adverse events associated with the use of osteoporosis medication is limited.^15^ For example, bisphosphonates (BP), first‐line anti‐osteoporosis medications, have been linked to rare but severe adverse events, including osteonecrosis of the jaw (ONJ) and subtrochanteric atypical femoral fractures (AFF). Those events could not be captured in the clinical trials or the extension trials, which tested the long‐term efficacy and safety of BPs (the FLEX study and HORIZON extension study) because those trials were not initially designed to investigate ONJ and AFF and the low incidence of those adverse events meant their statistical power was insufficient.^20^ Most of the evidence about ONJ and AFF was reported in observational studies utilizing large health care databases that had longer follow‐up and included larger numbers of people.^21^, ^22^

**Table 1:** The Difference Between Randomized Controlled Trials and Observational Studies

Randomized controlled trials	Observational studies
Proves causal inference—highest level of evidence	Complements findings from RCTs; able to demonstrate association, not causation
Randomization and blinding minimize confounding and other types of biases	Non‐randomized and susceptible to biases
High cost and short duration	Low cost and long follow‐up
Limited potential for study of rare and long‐term adverse events	Suitable for study of rare adverse events and long‐term outcomes
Small sample and strict inclusion criteria	Large sample and diverse patients
Limited generalizability	Reflects real‐world settings

Pharmaceutical manufacturers conduct RCTs (premarketing clinical trials) to investigate the therapeutic benefits and safety of new treatments before they get approved for marketing and prescribing by practitioners. Drawing on results from RCTs and the use of those drugs by the general population, postmarketing studies, which are observational in nature, are developed and have become essential to further study the effects of those new drugs in larger populations.^23^ Hence, observational studies can be used to complement findings from RCTs because they can use large sample‐sized patient populations that include clinically important subpopulations (eg, elderly, complex patients, and those exposed to polypharmacy), many of which might be excluded from randomized controlled trials^15^ (Table 1). For example, patients with severe chronic kidney disease (CKD) were excluded from most RCTs conducted for studying the effects of bisphosphonates. Therefore, the potential risks and benefits of those medications in this growing group of the population are unknown, and hence first‐line therapies (eg, bisphosphonates) are contraindicated in this relevant at‐risk population. Additionally, compliance issues usually observed in the community^24^ are unlikely to be present in RCTs, hence limiting the transportability of treatment effects from one setting to another.

Although RCTs provide the highest level of evidence, a few meta‐analyses comparing treatment results from RCTs and well‐designed observational studies across a range of clinical conditions have found that although discrepancies can occur, the estimates of treatment effects are similar.^25^, ^26^, ^27^ A retrospective cohort study comparing the real‐world effectiveness of osteoporosis medications risedronate to alendronate and calcitonin using an administrative claims database confirmed the fracture risk reduction at 6 and 12 months of risedronate and alendronate that was shown in RCT data and pooled post hoc analyses of the same treatments.^10^ The similarity of the results of the two differently designed studies suggests that observational studies can confirm and extend the results obtained from RCTs to a broader population.

Observational studies, as stated earlier, can be conducted in subpopulations that are usually excluded or underrepresented in RCTs. Hence, the results from those studies can validate the use of treatments in certain groups (Table 1). For example, high‐quality evidence supports the use of alendronate for prevention of vertebral fractures among glucocorticoid‐treated patients, but the quality of evidence is low for prevention of nonvertebral fractures; evidence is lacking for prevention of hip fracture because the RCTs were small and were not designed to study rare events such as hip fractures.^28^, ^29^ To close this evidence gap, a recent retrospective cohort study using a Swedish national database of 433,195 patients aged 65 or older investigated whether alendronate treatment in older patients using prednisolone (the primary glucocorticoid for long‐term treatment of inflammatory diseases) was associated with decreased hip fracture risk. The results showed that among older patients using medium to high doses of prednisolone, alendronate treatment was associated with a significantly lower risk of hip fracture over a median of 1.32 years.^30^

## Sources of Data for Pharmacoepidemiological Studies

The research question usually defines the type of data required for a certain study. Data sources are broadly classified into primary (actively collected) and secondary (routinely collected or existing) data sources.^31^ Important considerations for choosing data include whether the key variables are available to identify exposures, outcomes, and confounders. Data should be highly detailed, contain historical information to determine baseline patient characteristics, and represent an adequate duration of follow‐up.^31^

### Primary data sources

Primary data involve the collection of new data by the investigator directly from study participants with prospective follow‐up from a certain time point to the future/outcome of interest. They can be tailored to answer the exact research question by collecting specific exposure variables and therefore be more complete. However, prospective data collection has several drawbacks, including being time‐consuming, loss to follow‐up, hawthorne effect (change in behavior while being “observed”), and high cost.^32^

#### Prospective observational studies

These studies collect data from subjects and subsequently observe them over time for the effects of specific treatments on particular outcomes. A classic example of prospective cohort studies is the Framingham Heart Study (FHS), one of the first longitudinally followed large cohort studies initiated in 1948, which also has an ancillary study, the Framingham Osteoporosis Study.^33^ Another example is the Dubbo Osteoporosis Epidemiology Study (DOES), one of the longest‐running prospective observational cohort studies of osteoporosis in women and men internationally.^34^

#### Registries

Registries are also primary data sources that are systematically collected for research. Patients are typically identified when they present for care, and the data collected generally include clinical and laboratory tests and other information such as the length of their hospital stay and their socioeconomic status. Registries are defined by specific diseases/conditions, exposures (eg, to drug products), time periods, or populations.^31^ The Global Longitudinal Study of Osteoporosis in Women (GLOW) is a prospective registry study that involved forming an osteoporosis registry for women aged 55 years and older by collecting data from 10 countries over a five‐year period.^35^

### Secondary data sources: routinely collected health data

The growing trend of the development of large‐scale routinely collected health data in the past 20 years has resulted in an increase in the use of large patient information sources in pharmacoepidemiology.^36^ The two main types of databases available for observational studies are electronic medical records (produced as a result of clinical care) and administrative databases (by‐product of financial transactions).^37^ These databases have a lot of advantages, including their large size that allows the study of rare events, their representativeness, and their availability at relatively low cost and without long delays, which makes them accessible and efficient.^36^ Studies conducted using secondary data sources are usually conducted using readily available data, collected in the past. Because investigators do not have control over the process of data collection as in prospective studies, key variables required for the study might be unavailable in an otherwise ideal data set. Hence, linking different data sets or using already linked ones provides richness to the study information.^31^

A number of primary care records databases are available worldwide. Commonly used ones in the bone research field are the UK Clinical Practice Research Datalink (CPRD) and the Catalan Sistema d'Informació pel Desenvolupament de l'Investigació en Atenció Primària (SIDIAP). Their data have been linked to many other data sets to address questions about the risks and benefits of different treatments. In a recent example, the CPRD data set and SIDIAP have been linked to inpatient data, national renal registries, and mortality data to study the association between the use of oral bisphosphonates and a number of outcomes in patients with moderate‐severe chronic kidney disease (CKD). The findings from the UK data have been presented recently in the form of conference abstracts^38^, ^39^, ^40^ and are now being replicated in the Catalan data set for confirmation.

In other regions like Denmark and Sweden, excellent quality secondary care data are available. Given the existence of a unique person identifier throughout the health and social systems, patient data can be obtained from hospital (out‐ and inpatient) records, linked to pharmacy dispensations data, and sociodemographic information. Numerous pharmacoepidemiological studies have been conducted in the field of osteoporosis using such data, including a recent example where more than 60,000 users of alendronic acid were observed for up to 10 years to study the effects of long‐term bisphosphonate users on rare outcomes such as osteonecrosis of the jaw and femoral shaft fractures.^21^, ^22^

## Common Designs of Pharmacoepidemiological Studies

The goal of observational pharmacoepidemiological studies is to identify the association between different exposures or treatments and specific outcomes. Different study designs are used in pharmacoepidemiologic research (Table 2).^41^ Descriptive studies describe the distribution of disease or other health outcomes in populations, whereas analytical studies test hypotheses by studying the association between different factors and outcomes. However, two of the most commonly implemented study designs are cohort and case‐control studies, which offer the advantage of measuring the association between certain treatments and health outcomes with a temporal dimension.^32^

**Table 2:** Observational Study Designs in Pharmacoepidemiology

Descriptive observational studies	Analytical observational studies
1. Case report	1. Case‐control studies
2. Case series	2. Cohort studies
3. Ecologic studies	3. Hybrid studies
	a. Nested case‐control studies
	b. Case‐cohort studies
	c. Case‐crossover studies
	d. Case‐time studies
4. Cross‐sectional studies	

### Cohort studies

In cohort studies, participants are identified based on their drug exposure and followed over time for the rates of outcomes (incidence, relative risks). Cohort studies can be prospective or retrospective. Prospective cohort studies involve following up participants from the present time to the future. They are designed to actively collect data from participants and hence specific variables required to answer the research question can be obtained. However, they can be expensive to conduct, require long duration of follow‐up, especially for rare outcomes, and they are susceptible to loss to follow‐up.^42^ Retrospective cohort studies involve identifying participants based on their drug exposure and then looking into their past to examine certain outcomes. The main disadvantage of this study design is the limited control the investigator has over data collection.^43^ Table 3 lists the advantages and disadvantages of cohort studies.

**Table 3:** Advantages and Disadvantages of Cohort and Case‐Control Studies

Type of study	Advantages	Disadvantages
Cohort	Better in finding a causal link	Not suitable for rare diseases or diseases with long latency as large number of subjects required
	Suitable for rare exposures and examining multiple outcomes for one exposure	Problems with loss to follow‐up
	Prospective design (usually)	Requires long duration and is more expensive
	Estimation of absolute and relative risks	Susceptible to confounding by indication and immortal time bias
Case‐control	Suitable for rare outcomes or outcomes with long latency	Not suitable for rare exposures
	Quicker to conduct and lower costs than cohort studies	Difficult to find an appropriate control group
	No problem with loss to follow‐up	Cannot estimate risks
	Requires smaller sample size	Susceptible to recall and interviewer bias

### Case‐control studies

In case‐control studies, participants are identified based on their outcomes, cases are those who have the outcome while controls do not, and the past exposure to drug is compared between the two groups. They use retrospectively collected data and are best used to study rare outcomes and those with long latency period. In comparison to cohort studies, case‐control studies cannot estimate risks, are quick, less expensive to conduct, require a smaller sample, and can assess multiple drug exposures for one outcome.^43^, ^44^ Table 3 lists the advantages and disadvantages of case‐control studies.

## Atypical Femoral Fractures and Bisphosphonate Use in Pharmacoepidemiology

Long‐term bisphosphonate use has been linked to the occurrence of atypical femoral fractures (AFFs). AFF is a rare adverse event characterized by a noncomminuted, transverse fracture of the subtrochanteric or femoral shaft regions with specific radiological features.^45^ Studying AFFs is a good example to explore the different types of study designs and data sources that could be used in pharmacoepidemiology and how different analyses could yield mixed results. AFFs were first described in case series in 2005.^46^ This was followed by multiple case reports and case series,^47^, ^48^, ^49^ secondary analyses of results from randomized controlled trials and extension trials,^50^ cross‐sectional studies,^51^ prospective^52^ and retrospective cohort studies,^53^, ^54^ case‐control studies,^55^, ^56^ and hybrid studies.^22^, ^57^

Studies of AFFs and their association with bisphosphonates fall into two general categories. First are fractures identified using large registry or database approaches with International Classification of Diseases, 9th edition (ICD‐9), but no radiographic adjudication to confirm features of atypia.^58^ Most of those studies showed that atypical fracture rates have increased in patients exposed to bisphosphonates. However, typical and atypical fractures were not distinguished in these studies because original radiographs were not reviewed and hence AFFs rates were overestimated.^59^ Second are fractures ascertained by radiographs. Those studies showed that the absolute incidence of AFFs is relatively low, but there is a significant association between bisphosphonates and AFFs.^60^

## Opportunities for Pharmacoepidemiological Studies

As stated previously, conducting observational (cohort, case‐control, and others) studies has many advantages. Using large health care databases may include a wide variety of data because various forms of health care information can be linked. They include very large numbers of diverse patients (including those not typically recruited in RCTs because of comorbidity, sociodemographics, or logistics), registered in potentially any treatment center/s (including nonspecialized ones), with long follow‐up and at much lower cost.^14^ This enables the study of rare events and allows for generalizability of the results.^61^ Furthermore, some databases contain detailed information about drug use, including initiation and discontinuation data, reason/s for discontinuation, number of prescriptions, and dosing regimens. This allows for assessing treatment persistence and compliance and its effect on clinical outcomes in typical patient settings.^16^ In a recent example, a review of observational prospective and retrospective studies investigating the adherence, compliance, and persistence with osteoporosis therapies in North America and Europe indicated that low compliance and persistence rates for osteoporosis therapies in the real‐life setting results in increased rates of fragility fractures.^62^

## Challenges of Pharmacoepidemiological Studies

Although observational studies have numerous benefits and their findings can confirm and complement findings from RCTs, they also have a number of limitations which may prove challenging. As previously mentioned, a limitation of prospective cohort studies specifically is the long follow‐up period while waiting for events or diseases to occur. This design is inefficient for investigating diseases with long latency periods and is vulnerable to a high loss‐to‐follow‐up rate. In addition, those studies are expensive to conduct.^32^ Although retrospective studies using health care databases may be more practical, the information has not been collected specifically for research purposes and there are concerns about the accuracy and precision of the data used in those studies. For example, errors in data coding can result in misclassification of drug exposure and outcomes as well as diagnostic misclassification.^16^, ^36^, ^61^ In addition, the data are limited to available variables in the data source. Therefore, there may be issues with missing data elements and unmeasured confounders. Ensuring high‐quality data and integrity is vital for research studies; however, data quality and details of information differ substantially among health care databases worldwide.^16^, ^36^, ^61^ Fortunately, examples exist of databases where fractures have been validated with good accuracy, including both primary care (CPRD^63^ and SIDIAP,^64^, ^65^ amongst others) and secondary care (eg, Danish registries^66^) data.

In addition to the challenges outlined above, observational studies are associated with methodological issues, including different types of biases that can affect the internal and external validity of the study if they have not been adequately accounted for. These methodological issues are not as obvious as the ones previously described and may be the most challenging. The following section discusses some of the most important challenges that face researchers when conducting observational studies and techniques to address them, including *missing data*; *selection bias*, which is more commonly known in pharmacoepidemiology studies as *confounding by indication*; *immortal time bias* and *time‐varying confounding*, which are associated with changes in drug exposure and covariates during the follow‐up period; and *uncontrolled confounding*, also known as *residual confounding*, which is related to unmeasured confounders.

### Missing data

Using databases with large amounts of missing information that do not have rigorous and standardized data editing, cleaning, and processing procedures increases the risk of inconclusive and potentially invalid study results.^31^ Missing data is a frequent complication of any real‐world study. The causes of missingness are often numerous, some due to design and some to chance. Some variables may not be collected from all subjects, some subjects may decline to provide values, and some information may be purposely excised, for example to protect confidentiality.^67^

There are a few methods to deal with missing data. It is important to recognize which variables are missing and the pattern of their missingness. For the initial analysis, an analysis of non‐missing values can be performed, called a complete‐case analysis.^68^ However, this approach can reduce the sample size and hence the power of the study, as well as incur selection bias, as patients with measures available will (in actual practice) tend to be different from those without.

Multiple imputation is a general approach to the problem of missing data. It involves complex statistical modeling aimed to allow for the uncertainty about the missing data by creating several different plausible imputed data sets and appropriately combining results obtained from each of them. Multiple imputation analyses will avoid bias when missing is “at random” (ie, completely at random conditional on certain characteristics such as age or comorbidity) and when enough predictors of missingness and of missing values, as well as the outcome variable are included in the imputation model.^67^

### Confounding by indication bias

Clinicians usually prescribe medications based on multiple factors, including the severity of patients’ illness and their clinical, behavioral, and functional characteristics. If some of these factors are imbalanced between drug users and non‐users and independently associated with the study outcome, then failing to control for such variables can lead to confounding by indication bias.^36^ Confounding by indication can sometimes be minimized by design (eg, in case‐only studies) or controlled for at the analytical stage using statistical methods, including restriction, multivariable adjustment, stratification, weighting, or matching.^69^, ^70^

Propensity score (PS) analyses have become one of the most popular methods to control for confounding by indication. It is defined as the conditional probability of being treated given the patients’ characteristics. PS can thus be used to balance between‐group differences and hence reduce bias.^71^ When a PS is calculated, it can be used in adjustment, matching, stratification, or in inverse probability weighting. Matching on the propensity score takes several approaches, but all are centered on finding the nearest match of a treated individual to a comparison subject(s) based on the scalar propensity score.^72^ In a recent prospective study that examined the effect of osteoporosis medication on mortality risk using the DOES cohort, a propensity score matched set was created by matching each treated participant with a non‐treated participant with a similar propensity score or similar likelihood of being treated.^11^

### Immortal time bias and time‐varying confounding

Immortal time bias (ITB) is a common issue in pharmacoepidemiology. ITB occurs when the event of interest/outcome could not have occurred for a certain time span because of the chosen study design and/or data analysis methods.^73^ For example, if a subject is observed from the time he had a hip fracture (index date) but the exposure started at the date of a first prescription of oral bisphosphonate after the fracture, the period of time from the index date to the first prescription date is “immortal” for drug users, as the patient must be alive to become a user. This bias systematically overestimates the outcome rate in the untreated group, while underestimating the rate in the treated group, therefore creating the illusion that the drug (here, oral bisphosphonate) is preventive against the outcome of interest. To avoid this bias, all immortal time should be fully accounted for in the analysis. A potential solution at the design stage is the use of a time‐varying exposure, where drug users are considered as non‐users for the time that begins from index (hip fracture) to start (first prescription) date. After treatment initiation/exposure to drug, subjects are then classified as exposed/treated.^73^

In addition to time variations in treatment initiation, other covariates that affect treatment exposure and outcome may also vary throughout follow‐up, resulting in the so‐called time‐varying confounding. This type of confounding can partially be controlled for using the time‐dependent analyses, where patient characteristics for drug users should be considered at the index date for the “non‐user” time and then reevaluated before start date for the user time.^74^ Other advanced methods can be used to more accurately account for time‐varying confounding, including inverse probability treatment weighting^75^ and marginal structural equations, amongst others.^76^

### Residual confounding and unmeasured confounders

In some studies, incompletely controlled “residual” confounding can occur for several reasons. Some confounders for the exposure or outcome may not be identified or measured in routine practice.^77^ For example, bone mineral density (BMD) has been reported to be an independent determinant of fracture risk and all‐cause mortality.^78^, ^79^ However, observational studies using secondary data sources such as administrative databases or primary care medical records do not usually have BMD results. Hence, the inability to adjust for BMD as a confounder can provide inaccurate effect estimates. Furthermore, residual confounding might still persist after adjusting for the measured variables because of misclassification or measurement errors.^77^, ^80^ Residual confounding can threaten the validity of a study; therefore, it should be assessed and approaches to estimating its effect, including sensitivity analyses and instrumental variable (IV) approach, should be implemented.

The purpose of these sensitivity analyses is to make informed assumptions about the residual confounding and quantify its effect on the relative risk estimate of the drug‐outcome association. This is performed by varying the confounder prevalence in the exposed versus the unexposed and the magnitude of the confounder–disease association and obtaining different risk estimates over a wide range of parameter groupings.^77^, ^81^

Instrumental variable analyses rely on the existence of an “instrument,” a variable that has three key characteristics: 1) it is highly correlated with treatment; 2) it does not directly affect the outcome (other than through the effect of the treatment); and 3) it is not associated with potential (measured or unmeasured) confounders.^31^ One of the most common instruments in pharmacoepidemiology is the so‐called physician prescription preference, which is derived from the assumption that different providers or physicians have different preferences or treatment algorithms dictating how medications should be used.^82^ An example could be the choice of anti‐osteoporosis medication: the possibility that physicians strongly differ in their preference for different anti‐osteoporosis medications suggests that an IV defined at the level of the prescribing physician could be used to compare treatment effects. When the three assumptions above are fulfilled, IV analyses can account for confounding related to both measured and unmeasured variables.

## Conclusion

Pharmacoepidemiology is used extensively in osteoporosis research and involves the study of the use and effects of drugs in large numbers of people. Results from these studies can confirm and complement findings from RCTs and are more generalizable. Primary sources of data consist of prospective collection of new data and registries. Secondary data sources include medical records and administrative databases. These have a number of advantages, including large size, representativeness, the ability to study rare adverse events, and to measure and account for persistence and compliance in actual practice settings, at a much lower cost when compared with RCTs or primary data collection. Observational cohort and case‐control studies are the two most commonly implemented study designs in pharmacoepidemiology. Cohort studies can be prospective (tailored to answer the research question) and retrospective (already collected data), each of which has its strengths and limitations. Case‐control studies are suitable for studying rare outcomes and are cheaper to conduct. However, they are susceptible to recall bias and finding an appropriate control group could be challenging. Although conducting observational studies in pharmacoepidemiology has a lot of advantages, there are also a number of challenges. Missing data is a common complication of using databases, which is frequently dealt with using multiple imputation method. Confounding by indication arises when the decision to treat is driven by specific risk factors present more commonly in the treated group. Multivariable regression modeling and propensity score analyses amongst others can be used to minimize confounding. Immortal time bias and time‐varying confounding are other common sources of bias, which can be accounted for using time‐dependent analysis. Finally, residual confounding due to lack of information on key variables or measurement errors should be assessed, and sensitivity analyses and specific methods (eg, instrumental variables) should be considered. An understanding of these biases and how to best overcome them is essential to both carrying out and appraising these studies.

## Disclosures

The authors have no conflicts of interests to declare.
